# The three dimensional analysis of the Sforzesco brace correction

**DOI:** 10.1186/s13013-016-0092-9

**Published:** 2016-10-14

**Authors:** Sabrina Donzelli, Fabio Zaina, Monia Lusini, Salvatore Minnella, Stefano Respizzi, Luca Balzarini, Salvatore Poma, Stefano Negrini

**Affiliations:** 1 ISICO (Italian Scientific Spine Institute), Milan, Italy; 2 Humanitas Research Hospital, Milan, Italy; 3 University of Brescia, Brescia, Italy; 4 IRCCS Don Gnocchi, Milan, Italy

## Abstract

**Background:**

Scoliosis is a three dimensional deformity, and brace correction should be 3D too. There is a lack of knowledge of the effect of braces, particularly in the sagittal and transverse plane. The aim of this study is to analyse the Sforzesco Brace correction, through all the parameters provided by Eos 3D imaging system.

**Method:**

Design: This is a cross sectional study from a prospective database started in March 2003.

Participants: 16 AIS girls (mean age 14.01) in Sforzesco brace treatment, with EOS x-rays, at start, in brace after 1 month and out of brace after the first 4 months of treatment. *Outcome measures: All the parameters and the Torsio-Index obtained from 3D Eos System, in and out of brace, in the three planes.* Statistical analysis: *the variability of the parameters and the mean differences were analyzed and compared using paired T test. ANOVA was used for multiple comparisons. Critical P value was set at 0.05.*

**Results:**

In the comparison of in-brace vs start of treatment, the mean Cobb angle changed significantly from 36.44 +/− 4 to 28.99 + −3.9° (*p* = 0.01). Significant changes in all the sagittal parameters were found (*p* = 0.02). In the axial plane, the Torsio Index changed significantly in-brace for thoracolumbar and lumbar curves (*P* < 0.05). The analysis of the single vertebral tilt demonstrated that the effect of the brace is mostly concentrated at specific segments: T4-T5, T10-T12, L1 and L5 in the axial plane and T3-T6 and T10-L1 in the frontal plane.

**Conclusion:**

The Sforzesco brace mostly modifies the middle of the spine and preserves the sagittal balance. The single vertebral orientation in each plane should be considered together with the typically used values to assess brace effect.

## Background

**Fig. 1:**
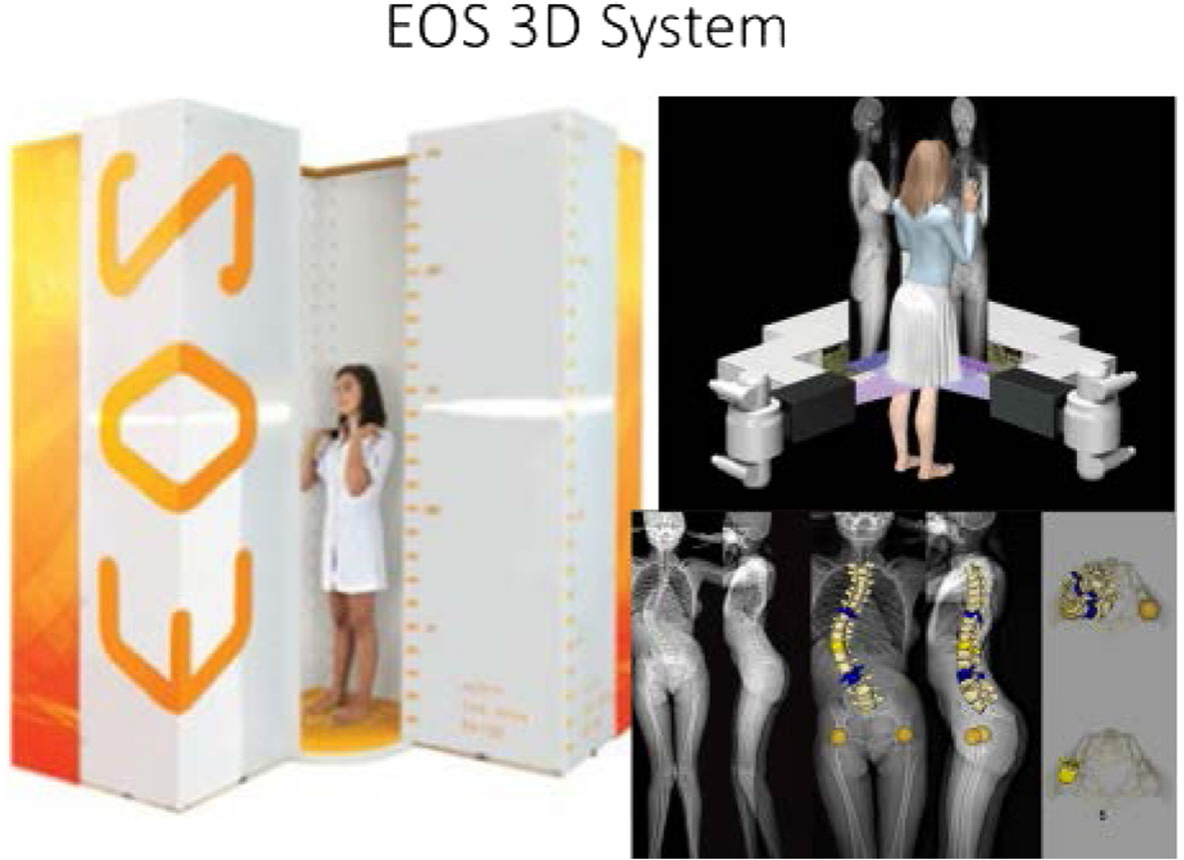
Show the EOS 3D System, and an example of the output of the 3D reconstruction

One of the major revolutions in the field of adolescent idiopathic scoliosis during the past 10 years is the development of 3D imaging devices in standing position, such as EOS Imaging. The EOS system is a new biplanar low-dose radiographic system [1]. Through the 3D reconstructions produced by dedicated user-friendly software, it is possible to calculate and visualize a series of regional and local parameters characterizing the spinal deformity [2]. This new technology allows the clinician to deepen the direct effect of braces in all three space planes [3]. The Fig. 1 shows an example of the output of the 3D reconstruction obtained through the EOS System.

Brace efficacy can play a role in determining the final outcome of a treatment [4]. Even if scoliosis is a three-dimensional deformity, the effect of braces has typically been analysed only in the coronal plane.

In more recent years, braces have become really three dimensional, adding a de-torsion action and addressing the whole shape of the trunk and its deformity [5]. The main expression of these new evolutions are the Rigo-Cheneau system [6, 7], the PASB brace [8], and the Sforzesco brace [9].

Different braces can act in different ways, and may act in one plane more than in the other. Therefore, personalized prescription of the brace can optimize treatment [10].

The aim of this study was to verify the immediate tri-dimensional in-brace correction and short term results (first 4 months of treatment) in a sample of patients treated with Sforzesco Brace. The secondary aim, was to evaluate if the 3D reconstruction was useful to verify and optimize brace efficacy.

## Methods

### Participants

Sixteen female patients treated with full-time Sforzesco brace, for adolescent idiopathic scoliosis (AIS) were included, according to the following inclusion criteria:

- AIS diagnosis.
- Sforzesco brace prescription 23 h/day,
- Availability of EOS, x-rays with 3D reconstruction at time 1 (start of treatment without brace), time 2 (1 month of brace wear and in-brace) and time 3 (after the first 4 months of therapy, out of brace).

### Design

This is a cross sectional study that was conducted in accordance with the Helsinki Declaration, and all the participants signed an informed consent to allow permission to use clinical data for research purposes.

### Outcome measures

- In-brace correction compared to the baseline characteristics (IN vs PRE).
- The effect of the brace after the first 4 months of therapy, with x-ray out of the brace, compared to the beginning of treatment (PRE vs OUT).
- Brace correction obtained after the first 4 months, out of brace, compared to the in-brace correction (IN vs OUT).

All the following outcomes were analyzed in the three planes, according to 3D EOS System reconstruction output:

- Sagittal parameters: Pelvic Incidence (PI), Pelvic Tilt (PT), Sacral Slope (SS), Thoracic Kyphosis 1 (TK T1-T12), Thoracic Kyphosis 2 (T4-T12), Lumbar Lordosis 1 (LL L1-L5), and Lumbar Lordosis 2 (LL L1-S1).
- Single intervertebral orientations in the horizontal, lateral and frontal planes.
- Axial vertebral rotations of the apex of the curve and the end vertebrae.
- Torsion-Index: This is an index proposed by Steib [11]. It is the mean of two sums of intervertebral axial rotations from the lower junction to the apex and then from the apex to the upper junction [12].

### Statistics

The Shapiro-Wilk test was applied to test data for normality. The paired *t*-test and the Wilcoxon signed-ranks test were used to test variability PRE-IN, IN-OUT and PRE-OUT. One-way ANOVA was used for multiple comparisons among single intervertebral orientations. The critical P value was set at 0.05.

## Results

The Cobb angle correction for the in-brace measurement was 10.30 +/− 7.60° (CI 95 % 6.30-14.33) (*p* = 0.001). When the brace was removed, part of the correction was lost, as expected, (−5.72 +/− 5.50°) (*p* = 0.0008) but a good average correction is maintained if compared to the starting point: (−4.60 +/− 7.51°) (*p* = 0.02) as shown in Table 1.

**Table 1:** show the average Cobb angle correction: PRE = average cobb angle at start; IN = average cobb angle at 1 month measured during brace wear; OUT = average cobb angle at the x-ray made after 4 months of therapy without the brace

Outcome	Pre Mean (SD)	IN Mean (SD)	OUT Mean (SD)	PRE-IN (SD)	PRE-OUT (SD)	OUT-IN (SD)
Cobb angle	38.20 (15.42)	27.90 (15.05)	33.61 (16.90)	10.30 (7.60)	5.72 (5.50)	4.60 (7.51)

Lumbar Lordosis is significantly reduced by the brace, and this reduction is maintained OUT of brace. Thoracic Kyphosis (T4-T12) showed a slight decreasing trend during treatment, while there were no changes in the Thoracic Kyphosis measured at T1-T12. Table 2 shows the results in the sagittal plane. There are some transient changes of the remaining sagittal parameters, but they are not maintained after brace removal.

**Table 2:** Sagittal parameters, PI = pelvic incidence, SS = sacral slope; PT = pelvic tilt; TK = thoracic Kyphosis; LL = lumbar lordosis. TK1 = measured from t1 to t12 TK2 = measured from t4 to t12; LL1 = measured from L1 to L5 and LL2 = lumbar lordosis measured from l1 to s1

	PRE-IN	*p*	PRE-OUT	*p*	OUT-IN	*p*
PI	5,3	0.04	3,8	N.S.	1,5	0.008
SS	3,4	0.003	−2,8	N.S.	6,1	0.005
PT	−3,8	0.023	1,4	N.S.	−5,2	0.001
TK 1	5,7	0.01	2,7	0.04	3,1	N.S.
TK 2	−0,4	N.S.	2,1	N.S.	−2,5	N.S.
LL 1	4,7	0.0005	0,4	N.S.	4,3	0,0005
LL 2	7,6	0.0008	1,1	N.S.	6,4	0.0008

No significant differences were found for the Torsion Index. When evaluating the intervertebral orientations, significant changes were found only in the coronal plane, induced only during brace wear, and for only levels T4 and T5, and from T10 to L1.

## Discussion

According to these results, the Sforzesco brace has its strongest influence on the middle of the spine in the axial plane, while the Torsion Index cannot be considered an index of brace correction. The effectiveness of the Sforzesco brace is confirmed again [9]. This study also showed that there is a slight trend towards straightening of the spine during brace treatment. Kyphosis reduction and flat back are predictors of scoliosis progression [13], but the Sforzesco brace seems to slow down the progression of curves in the sagittal plane.

The three-dimensional elongation effect, which is typical of the Sforzesco brace, can be responsible for the main effect focused on the middle part of the spine, and seen in the axial plane. This is a preliminary study, which offers some interesting insight into the mechanics of the Sforzesco brace correction, but the interpretation of these results must be done carefully. The main limitation is a very small sample size, associated with a large heterogeneity of data which threaten the internal validity of the study. In fact, this small sample included patients with very severe curves mixed with those who had milder ones, as demonstrated by the mean Cobb angle and the standard deviation. In addition, the included patients have different ages and level of bone maturation, therefore it is not possible to discuss the amount of the correction obtained. The amount of the in-brace correction, in terms of a percentage of correction, depends not only to the effect of the brace, but also on the curve morphology, the curve magnitude at start, and the level of bone maturity. The lack of distinction in curve types, curve magnitude, bone maturity and age threaten the external validity too.

The operator measurement error, for EOS technology must be taken into account, and further studies are needed to evaluate the reliability of these new technologies. Comparison of different braces are needed not only to compare results, but also to improve the immediate in-brace correction, and EOS imaging can be a very useful tool for these purposes.

## Conclusion

The Sforzesco brace mostly effects the middle of the spine, and preserves the sagittal balance of the spine and pelvis. The single vertebral orientation in each plane can be very useful to better discriminate the effect of brace, and should be considered together with the maximum curvatures and the upper, apex and lower limit of each curve. Further studies are needed to take advantage of this new 3D imaging technology.
